# Meteorological Table for the Month of February, 1852

**Published:** 1852-04

**Authors:** Lawrence Young


### METEOROLOGICAL TABLE,

FOR THE MONTH OF FEBRUARY, 1852.

BY LAWRENCE YOUNG, ESQ.

| Day of month,...... | Morning,........ | 2 o'clock, P. M.,..... | Evening,........ | Mean temperature, | Barometer,........ | Rain-guage,........ | Course of wind,...... | REMARKS. |
|---|---|---|---|---|---|---|---|---|
| 1 | 34 | 35 | 33 | 34 | 29.20 | | w. | Variable. |
| 2 | 31 | 37 | 31 | 33 | 29. 0 | | n. | Cloudy. |
| 3 | 32 | 48 | 39 | 40 | 29.28 | | s. | Clear. |
| 4 | 37 | 58 | 45 | 47 | 29.10 | | e. | Variable. |
| 5 | 33 | 52 | 48 | 44 | 29.14 | 07 | e. | Cloudy. |
| 6 | 55 | 55 | 48 | 53 | 28.92 | 28 | w. | Do. |
| 7 | 28 | 45 | 39 | 37 | 29.31 | | n. w. | Variable. |
| 8 | 28 | 46 | 36 | 37 | 29.37 | | s. s. e. | Clear. |
| 9 | 23 | 51 | 43 | 39 | 29.25 | | s. | Do. |
| 10 | 44 | 50 | 50 | 48 | 29.— | 81 | s. s. w. | Cloudy. |
| 11 | 32 | 31 | 30 | 31 | 28.87 | 31 | w. s. w. | Do. |
| 12 | 23 | 34 | 29 | 29 | 29.15 | | w. n. w. | Clear. |
| 13 | 40 | 50 | 38 | 42 | 29.03 | | w. s. w. | Variable. |
| 14 | 24 | 37 | 33 | 31 | 29.38 | | s. w. | Clear. |
| 15 | 34 | 43 | 41 | 39 | 29.10 | 07 | s. s. w. | Cloudy. |
| 16 | 31 | 53 | 47 | 44 | 29.04 | | swly. | Clear. |
| 17 | 26 | 40 | 35 | 33 | 29.05 | | w. | Do. |
| 18 | 25 | 35 | 31 | 30 | 29.27 | | n. | Variable. |
| 19 | 25 | 40 | 35 | 33 | 29.41 | | e. n. e. | Do. |
| 20 | 30 | 32 | 32 | 31 | 29.48 | 54 | e. n. e. | Cloudy. |
| 21 | 26 | 47 | 49 | 41 | 29.26 | 1.00 | e. | Do. |
| 22 | 45 | 50 | 47 | 47 | 29.10 | 50 | w. | Do. |
| 23 | 36 | 46 | 43 | 42 | 29.04 | 10 | s. w. | Variable. |
| 24 | 41 | 68 | 62 | 57 | 28.80 | | s. w. | Clear. |
| 25 | 37 | 44 | 37 | 39 | 28.83 | | n. | Do. |
| 26 | 26 | 42 | 37 | 35 | 29.16 | | w. | Variable. |
| 27 | 32 | 43 | 42 | 39 | 29.23 | | e. | Do. |
| 28 | 48 | 44 | 30 | 41 | 28.85 | 1.09 | n. w. | Cloudy. |
| 29 | 15 | 37 | 34 | 29 | 29.29 | | e. n. e. | Clear. |
| | | | | 38.8 | | 4.77 | | |

